# Interfacial reaction control and its mechanism of AlN epitaxial films grown on Si(111) substrates by pulsed laser deposition

**DOI:** 10.1038/srep11480

**Published:** 2015-06-19

**Authors:** Wenliang Wang, Weijia Yang, Zuolian Liu, Haiyan Wang, Lei Wen, Guoqiang Li

**Affiliations:** 1 State Key Laboratory of Luminescent Materials and Devices, South China University of Technology, Wushan Road, Guangzhou 510640, China; 2 Department of Electronic Materials, School of Materials Science and Engineering, South China University of Technology, Wushan Road, Guangzhou 510640, China

## Abstract

High-quality AlN epitaxial films have been grown on Si substrates by pulsed laser deposition (PLD) by effective control of the interfacial reactions between AlN films and Si substrates. The surface morphology, crystalline quality and interfacial property of as-grown AlN/Si hetero-interfaces obtained by PLD have been systemically studied. It is found that the amorphous SiAlN interfacial layer is formed during high temperature growth, which is ascribed to the serious interfacial reactions between Si atoms diffused from the substrates and the AlN plasmas produced by the pulsed laser when ablating the AlN target during the high temperature growth. On the contrary, abrupt and sharp AlN/Si hetero-interfaces can be achieved by effectively controlling the interfacial reactions at suitable growth temperature. The mechanisms for the evolution of interfacial layer from the amorphous SiAlN layer to the abrupt and sharp AlN/Si hetero-interfaces by PLD are hence proposed. This work of obtaining the abrupt interfaces and the flat surfaces for AlN films grown by PLD is of paramount importance for the application of high-quality AlN-based devices on Si substrates.

Recently, aluminum nitride (AlN) has been considered to be one of the most promising materials for the applications of optoelectronic devices due to its excellent physical and chemical properties^1^,^2^,^3^. To date, AlN films have been prepared on Si substrates, due to the advantages of Si substrates, such as large-size wafers, high crystalline quality, electric and thermal conductivities, and low cost, *etc*^4^,^5^,^6^. So far, AlN films grown by metal organic chemical vapor deposition (MOCVD) and molecular beam epitaxy (MBE) have been used in the application in thin films bulk acoustic wave resonators (FBARs) and surface acoustic wave (SAW) devices, *etc*^7^,^8^,^9^,^10^. It is known that the performance of AlN-based devices is dependent on the properties of interfaces and surfaces of as-grown AlN films^11^,^12^,^13^. AlN films grown on Si substrates by MOCVD and MBE technologies usually require high temperature growth^14^,^15^, which would lead to the formation of interfacial layer between films and Si substrates owning to the interfacial reactions between AlN films and Si substrates and is detrimental to the subsequent growth. Therefore, to obtain sharp and abrupt interfaces and smooth films surfaces, a low temperature growth is of critical importance. Nowadays, pulsed laser deposition (PLD) technology has been deployed to grow AlN films on various substrates at a very low temperature^16^,^17^,^18^, since the pulsed laser can supply high energy for the migration of precursors when arriving at substrates. This can effectively suppress the serious interfacial reaction between films and substrates, and lead to the formation of sharp and abrupt interfaces^19^,^20^,^21^. Furthermore, although there are several works report on the study of AlN/Si hetero-interface grown by PLD^11^,^12^, most of them focus on the study of the interfacial properties. The mechanisms of interfacial reactions between AlN films and Si substrates lack thorough study.

In this work, we report on the epitaxial growth of high-quality AlN films on Si(111) substrates by PLD through controlling the interfacial reactions between AlN films and Si substrates, and systematical study on the revolution of AlN/Si hetero-interfaces from amorphous to sharp and abrupt interface, as well as their corresponding mechanisms. The as-grown AlN epitaxial films are studied by *in-situ* reflection high energy electron diffraction (RHEED), high-resolution X-ray diffraction (HRXRD), scanning electron microscopy (SEM), atomic force microscopy (AFM), and high-resolution transmission electron microscopy (HRTEM) for structural property and surface morphology. This work provides an important guidance for the growth of high-quality AlN epitaxial films on Si substrates by PLD, and sheds light on the future application of AlN-based devices on Si substrates.

The as-received 2-inch Si(111) substrates were cleaned by H_2_SO_4_ : H_2_O_2_ : H_2_O (3:1:1) and buffered-oxide-etch (BOE) HF (20:1) to obtain an oxide-free and hydrogen terminated Si surface. Subsequently, the as-cleaned Si(111) substrates were degassed in a ultra-high vacuum (UHV) load-lock with a background pressure of 1.0 × 10^−8^ Torr, and then were transferred into a UHV growth chamber with a background pressure of 3.0 × 10^−10^ Torr. Before the epitaxial growth, the as-degassed Si(111) substrates were taken a 60 min annealing process at 850 °C to remove the residual surface contaminations and achieve an atomically flat Si (111) surface for the subsequent deposition. During the epitaxial growth, the AlN films with various thickness ranging from ~6–300 nm-thick were grown by a KrF excimer laser light (λ = 248 nm, t = 20 ns) ablating the high-purity AlN (4 N) target in a nitrogen ambient under the pressure of 8 mTorr with the temperature of 700–850 °C. The energy density of the laser was set at 3.0 J.cm^−2^ with a pulse repetition of 30 Hz. A schematic diagram for AlN films grown on Si(111) substrates by PLD is shown in Fig. 1. The surface morphology, crystalline quality and interfacial property of as-grown AlN films were characterized by *in-situ* RHEED, SEM (Nova Nano SEM 430 Holland), AFM (Bruker Dimension Edge, American), HRXRD (Bruker D8 X-ray diffractometer with Cu Kα1 X-ray source *λ* = 1.5406 Å), and HRTEM (JEOL 3000F). As for the TEM characterization, AlN films grown on Si(111) substrates were made by mechanical polishing followed by low-energy and low-angle ion milling (Fischione 1010 Low Angle Ion Milling & Polishing System), ending up with the sample edge thickness of about 20 nm. The cross-section samples were then put into a JEOL 3000F field emission gun TEM working at a voltage of 300 kV, which gives a point to point resolution of 0.17 nm. The electron energy loss spectroscopy (EELS) attached to the TEM was deployed to evaluate the concentration distribution along the AlN/Si hetero-interfaces.

The RHEED measurement is adopted to monitor the growth process during the whole course. Figure 2a shows the photograph of AlN films grown at 750 °C on Si substrates, and Figs. 2b–f reveal the RHEED patterns of AlN films grown at various growth temperatures. It is clear that sharp and streaky patterns can be identified in Si[1–10] direction after 60 min annealing process at 850 °C, as shown in Fig. 2c, which is in striking contrast to that before the annealing process, as shown in Fig. 2b. Subsequently, AlN film is grown on as-annealed Si substrate. After growth of ~6 nm-thick films at high temperature of 850 °C, one can hardly identify the RHEED patterns, indicating that the very poor films are found, as illustrated in Fig. 2d. As for ~6 nm-thick AlN films grown at 750 and 700 °C, very clear AlN RHEED patterns can be found, as shown in Figs. 2e and f respectively. Moreover, the RHEED patterns for ~6 nm-thick AlN films grown at 750 °C are slightly clearer than those of ~6 nm-thick AlN films grown at 700 °C. Further increase in thickness of AlN films at 850 °C leads to the spotty RHEED patterns, as shown in Fig. 2g. This means that single-crystalline AlN films with relatively rough surfaces are grown. If the AlN films are grown at low temperatures (800-700 °C), the single-crystalline AlN films can also be obtained. For example, Fig. 2h shows the sharp and streaky RHHED patterns of ~300 nm-thick AlN films grown at 750 °C along AlN [11–20] direction. In this regard, an in-plane epitaxial relationship between AlN films and Si substrates is determined to be AlN[11–20]//Si[1–10]^22^,^23^,^24^.

SEM and AFM measurements are deployed to further investigate the surface morphology of as-grown AlN films. Generally, the surface roughness of films is usually determined by the root-mean-square (RMS) of roughness of films surface^25^. In this work, we use AFM measurement to study the different places of each sample to get the surface roughness value. Figure 3a reveals a SEM image for ~300 nm-thick AlN films grown at 850 °C, where very rough surface with a RMS surface roughness of 5.1 nm is obtained, Fig. 4a. The height profiles along a straight line on the surface are shown in Fig. 4b. When the growth temperature is decreased, the surface roughness of as-grown AlN films is gradually decreased. Especially, when the ~300 nm-thick AlN films are grown at 750 °C, very smooth AlN surfaces are grown with a surface RMS of 1.3 nm, as shown in Figs. 3b and 4c. The height profiles along a straight line on the surface are supplied in Fig. 4d. However, as the growth temperature of AlN films is further decreased to 700 °C, the surface morphology of as-grown ~300 nm-thick AlN films becomes poorer with several big islands and the RMS surface roughness of these as-grown films is measured to be 2.5 nm, Figs. 3c and 4e. The height profiles along a straight line on the surface are provided in Fig. 4f. We attribute the poor surface of AlN films grown at 850 °C and 700 °C to the formation of interfacial layer in the AlN films, which is detrimental to both the nucleation and coherence of AlN films during the growth^12^,^16^, and thereby leads to the poor surface morphology of AlN films with many grains ultimately. Fig. 3d is a cross-sectional SEM for AlN films grown at 750 °C for 90 min, in which ~300 nm-thick AlN films can be clearly indentified. Therefore, the growth rate for AlN films grown on Si substrates at 750 °C is about 200 nm/h. Evidently, the growth temperature plays an important role in growing high-quality AlN films. Meanwhile, the surface morphology for AlN films obtained this work with a RMS surface roughness of 1.3 nm is much smoother than that of AlN films grown on sapphire, W, and Ni substrates^19^,^20^,^21^,^24^.

Figure 5a illustrates a typical XRD 2*θ-ω* scans for AlN films grown on Si substrates at temperature ranging from 700 to 850 °C. The peaks observed at 2*θ* = 28.01°, 58.85° and 95.07 ° are the diffractions from Si(111), Si(222) and Si(333), respectively; while the peaks located at 2*θ* = 36.02° and 76.42° are ascribed to AlN(0002) and AlN(0004), respectively^12^,^26^. In this regard, the out-of-plane epitaxial relationship between AlN and Si is of AlN(0001)//Si(111).

The *φ* scan is that rotation of the sample along the *φ* axis (usually in the plane of the sample) in XRD measurement^27^. Meanwhile, the *φ* scan is a typical method to identify whether the films contain twins and the rotational symmetry of asymmetry plane both for films and substrates^27^,^28^,^29^. Apparently, the combination of 2*θ*-*ω* and *φ* scans can help us to determine whether the films are single-crystalline and the in-plane relationship between the films and the substrates. Figure 5b shows *φ* scans of Si(224) and AlN(10–15), where three-fold rotational peaks separated by 120° for Si(224) and six-fold rotational peaks with an interval of 60° for AlN(10–15) are clearly identified. Therefore, the in-plane epitaxial relationship between AlN and Si is determined to be AlN[11–20]//Si[1–10] with a lattice mismatch of 15.9%, as shown in Fig. 5c30^30^.

The crystalline quality of as-grown ~300 nm-thick AlN films is studied by X-ray rocking curves (XRCs). As we know, the full-width at half-maximums (FWHMs) of XRCs are related to the dislocation density in as-grown AlN films. The temperature dependence of the FWHMs for ~300 nm-thick AlN films grown on Si(111) substrates at temperatures ranging from 700 to 850 °C is shown in Fig. 5d. It can be noted that the FWHMs for AlN(0002) and AlN(10–12) are 2.5° and 2.2°, respectively, at the growth temperature of 850 °C and then the FWHMs for AlN(0002) and AlN(10–12) are monotonously decreased when the growth temperature is decreased from 850 to 750 °C . Whereas if the ~300 nm-thick AlN films are grown at 750 °C, the FWHMs for AlN(0002) and AlN(10–12) reach the minimum values of 0.6° and 0.8°, respectively. However, if we further decrease the growth temperature, the crystalline quality of as-grown ~300 nm-thick AlN films becomes slightly poorer with FWHMs for AlN(0002) and AlN(10–12) of 0.8° and 1.0° at 700 °C, respectively. Conclusively, the growth temperature makes a significant impact on the crystalline quality of AlN films. The tendency of the growth temperature of crystalline is well consistent with that of surface morphology. Furthermore, the quality of AlN films obtained in this work is much better than that grown on sapphire substrates and is comparable with that of AlN films grown on Al substrates^16^,^17^,^24^. This achievement of high-quality AlN films is attributed to the utilization of PLD and low temperature growth. On the one hand, PLD can supply enough energy for the migration of AlN precursors on Si substrates, and thereby make the AlN growth at low temperature possible. On the other hand, the low temperature growth not only can effectively suppress the interfacial reactions between AlN films and Si substrates, but also is much easier for the nucleation of AlN films and causes the formation of less dislocation, which is in striking contrast to the high temperature growth of AlN films on Si substrates by MOCVD. These two aspects result in the growth of high-quality AlN films on Si substrates despite of the 15.9% lattice mismatch.

The grazing incidence X-ray reflectivity (GIXR) measurement is introduced to study the interfacial property of AlN/Si hetero-interfaces. Due to the requirement of GIXR, the thin film thickness should usually be <100 nm^27^,^31^. Therefore, we deploy ~30 nm-thick films for this study. Figure 6a is a GIXR and its simulated curves for AlN films grown on Si(111) substrates at 750 °C. Before the simulation, we assume that there are three layers, *i.e*., Si substrate, interfacial layer, and AlN film layer existing in the as-grown AlN films on Si(111) substrates, and the initial parameters of each individual, such as thickness, RMS surface roughness, RMS interfacial roughness *etc.,* also need to be input to get an initial simulation curve^12^,^21^. Subsequently, the initial simulation curve is fitted to the experimental curve by using the Fresnel equation integrated in TEPTOS software^12^,^27^. When the simulation curve well agrees with the experimental one, we eventually get the real parameters of each individual layer. In this way, we obtain that there is no interfacial layer existing between AlN films and Si substrates. This may be ascribed to the suppression of interfacial reactions between AlN films and Si substrates due to the effective suppression of diffused active Si atoms from the substrates which may react with AlN precursors^19^,^20^,^21^. Meanwhile, the fitting results from the simulation also reveal that the thickness of as-grown AlN films is 29.3 nm with a RMS surface roughness of 1.3 nm.

The temperature dependence of interfacial layer thickness is also conducted in this study, Fig. 6b. We find that the interfacial layer thickness is 8.0 nm at the growth temperature of 850 °C, and is gradually decreased as the decrease in the growth temperature. Especially, when the growth temperature is 750 °C, the interfacial layer thickness reaches its minimum value of 0 nm. However, if the growth temperature is further decreased to 700 °C, the interfacial layer thickness becomes 1.5 nm. We attribute these results to the following^32^,^33^. At higher growth temperature of 850 °C, severe interfacial reactions take place between Si substrates and AlN films during the initial growth, leading to the formation of the 8.0 nm-thick interfacial layer, which is bad for the growth of subsequent growth of AlN films. If the growth temperature is decreased, the interfacial reactions become slower, and the interfacial layer thickness is greatly reduced. Particularly, when AlN films are grown at 750 °C, the interfacial reactions are effectively suppressed, and the sharp and abrupt interfaces can be therefore obtained^19^,^20^,^21^. However, the further decrease in growth temperature deteriorates the migration of AlN films on the substrates, and leads to the formation of 1.5 nm-thick AlN interfacial layer.

In order to systematically study the properties of the interfacial layer, TEM and EELS are deployed. Figure 7a shows the cross-sectional TEM and the corresponding fast Fourier transform (FFT) images for AlN films grown at 850 °C. We can find that there is an 8 nm-thick amorphous layer existing between AlN films and Si substrates. The EELS measurement is a normal method for detecting the atom concentration distribution, and has been widely used to check the concentration distribution in the interfacial layer^14^,^33^,^34^,^35^. When the EELS measurement is preformed across a certain distance of interfacial layer, the concentration distribution of atom along the interfacial layer can be detected. We then can identify the transition of the atoms from the curve, and therefore help us to find out the exact materials of interfacial layer^14^,^33^,^34^,^35^. In this regard, the EELS measurement is introduced to study the elements in AlN/Si interfacial layer. From the EELS curves, as shown in Fig. 7b, one can find that when the distance is away from the substrates, the Si concentration gradually decreases while the Al and N concentrations monotonously increase. Based on these results, we can therefore conclude that this interfacial layer is SiAlN layer^14^,^33^,^34^,^35^. The formation of this amorphous SiAlN layer may be ascribed to the reactions between Si atoms from Si substrates and AlN precursors produced by pulsed laser when ablating the AlN target during the high growth temperature. The formation of this amorphous SiAlN layer releases the stress between AlN and Si and causes the annihilation of dislocations^36^,^37^,^38^,^39^,^40^. However, the amorphous SiAlN layer is quite difficult for the nucleation of AlN films, and thus the AlN films grown on this amorphous SiAlN layer are of poor-quality. The AlN films grown at 750 °C show sharp and abrupt interfaces with no interfacial layer, as shown in Fig. 7c, which is in striking contrast to that of AlN films grown 850 °C. At this growth temperature, the diffused active Si atoms from substrates are effectively suppressed^19^,^20^,^21^, and therefore the suppression of interfacial reactions between AlN films and Si substrates is achieved. These sharp and abrupt AlN/Si hetero-interfaces benefit the subsequent growth of high-quality AlN films. Selected area electron diffraction (SAED) patterns are used to further study the AlN/Si hetero-interfaces, as shown in Fig. 7d. The in-plane relationship of AlN[1–100]//Si[11-2] can be identified^33^,^41^,^42^, which also confirms the high-quality AlN films grown at 750 °C. However, if the growth temperature is further decreased, one can find that there is a 1.5 nm-thick interfacial layer existing between AlN films and Si substrates, as shown in Figs. 7e and f, which is determined to be AlN layer using the same method mentioned above^14^,^33^,^34^,^35^. This 1.5 nm-thick AlN interfacial layer is under tensile stress, and therefore the lattice parameter for this AlN interfacial layer is quite different from those of AlN films on the topside shown in Fig. 7e. These results are well consistent with the GIXR measurement.

From these detailed studies on the properties of AlN films grown on Si substrates with various growth temperatures, we can obtain the growth mechanisms for AlN films grown on Si substrates by PLD. As for the AlN films grown on higher growth temperature of 850 °C, serious interfacial reactions take place between Si atoms diffused from Si substrates and AlN precursors produced by pulsed laser ablating the AlN target, leading to the formation of amorphous SiAlN interfacial layer. Although the formation of this amorphous SiAlN layer releases the stress between AlN and Si and causes the annihilation of dislocations from the substrates^36^,^37^,^38^,^39^,^40^, the amorphous SiAlN layer is quite difficult for the nucleation of AlN films, and thus the AlN films grown on this amorphous SiAlN layer are of poor-quality. This process is illustrated in Fig. 8a. As for AlN films grown on Si substrates at 750 °C, the interfacial reactions between Si substrates and AlN films have been effectively suppressed, which results in the formation of sharp and abrupt interfaces and eventually leads to the high-quality AlN films with small FWHMs and smooth surface, as shown in Fig. 8b. However, as the growth temperature is further decreased to 700 °C, 1.5 nm-thick AlN interfacial layer is formed to release lattice mismatch between AlN and Si during the initial growth. This interfacial layer contains fewer dislocations, which extend into the subsequent growth AlN films, as shown in Fig. 8c. In short, high-quality AlN epitaxial films grown on Si substrates can be obtained with suitable growth temperature by PLD.

High-quality AlN films with sharp and abrupt interfaces have been grown on Si substrates by PLD. It is revealed that the ~300 nm-thick AlN films grown on Si substrates at 850 °C show poor crystalline quality with the FWHMs for AlN(0002) and AlN(10–12) of 2.5° and 2.2°, quite rough surface with a RMS surface roughness of 5.1 nm, and a 8.0 nm-thick amorphous SiAlN layer. If the growth temperature is decreased from 850 to 750 °C, the quality of as-grown ~300 nm-thick AlN films is improved significantly. Especially, the as-grown ~300 nm-thick AlN films grown at 750 °C show high-quality with the FWHMs for AlN(0002) and AlN(10–12) of 0.6° and 0.9°, very smooth surface with a RMS surface roughness of 1.3 nm, and sharp and abrupt interface. However, when the growth temperature is further decreased to 700 °C, the quality of as-grown AlN films becomes slightly poorer. The FWHMs for AlN(0002) and AlN(10–12) are raised to 0.8° and 1.0°, and the RMS surface roughness and the thickness of interfacial layer are increased to 2.5 and 1.5 nm, respectively. The mechanisms for the evolution of interfacial layer from the amorphous SiAlN and layer to the abrupt and sharp AlN/Si hetero-interfaces by PLD are hence proposed. This work of obtaining the abrupt interfaces and the flat surfaces for AlN films grown by PLD is of great importance for the application of high-quality AlN-based devices on Si substrates.

## Additional Information

**How to cite this article**: Wang, W. *et al*. Interfacial reaction control and its mechanism of AlN epitaxial films grown on Si(111) substrates by pulsed laser deposition. *Sci. Rep*. **5**, 11480; doi: 10.1038/srep11480 (2015).

## Figures and Tables

**Figure 1:**
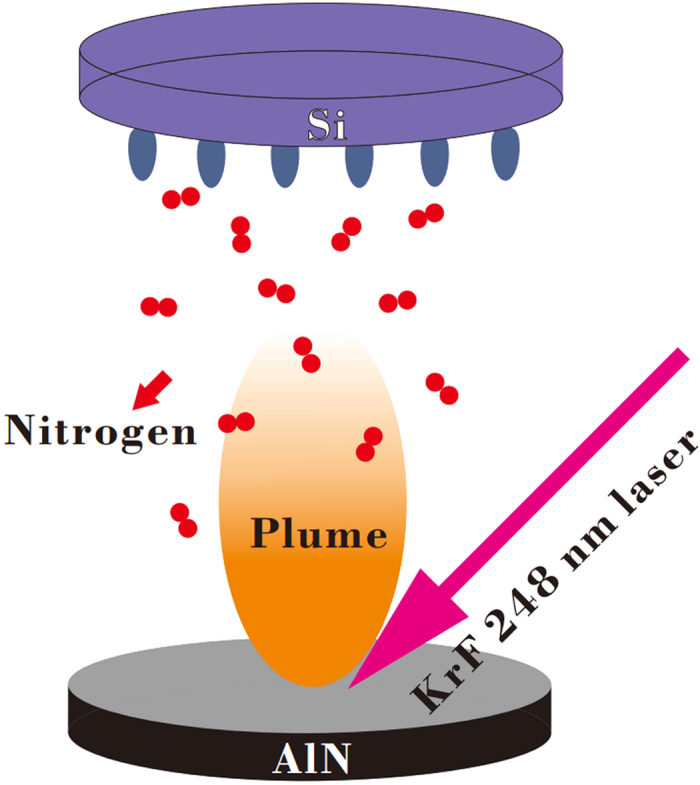
A schematic diagram for the epitaxial growth of AlN films on Si (111) substrates by PLD.

**Figure 2:**
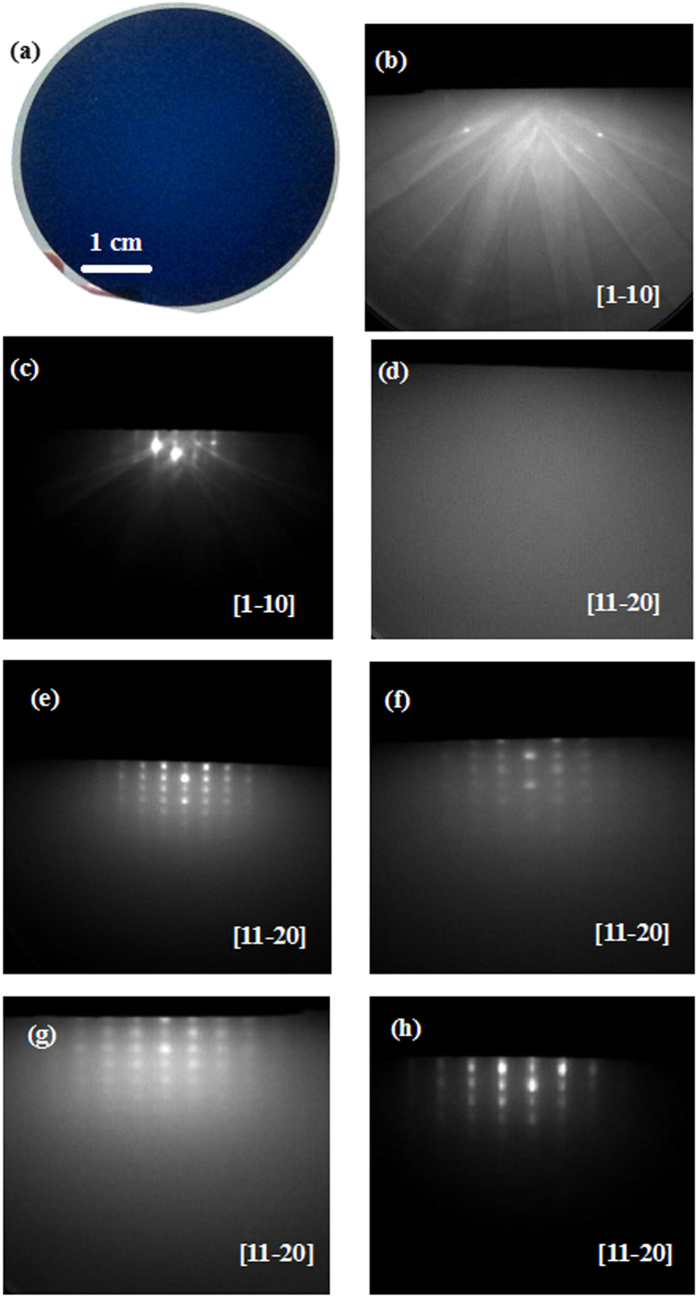
(**a**) Typical photograph of AlN film grown on Si substrate at 750 °C. RHEED patterns for Si substrates (**b**) before annealing process, and (**c**) after 60 min annealing process. RHEED patterns for ~6 nm-thick AlN films grown at (**d**) 850, (**e**) 750, and (**f**) 700 °C. RHEED patterns for ~300 nm-thick AlN films grown at (**g**) 850, and 750 °C.

**Figure 3:**
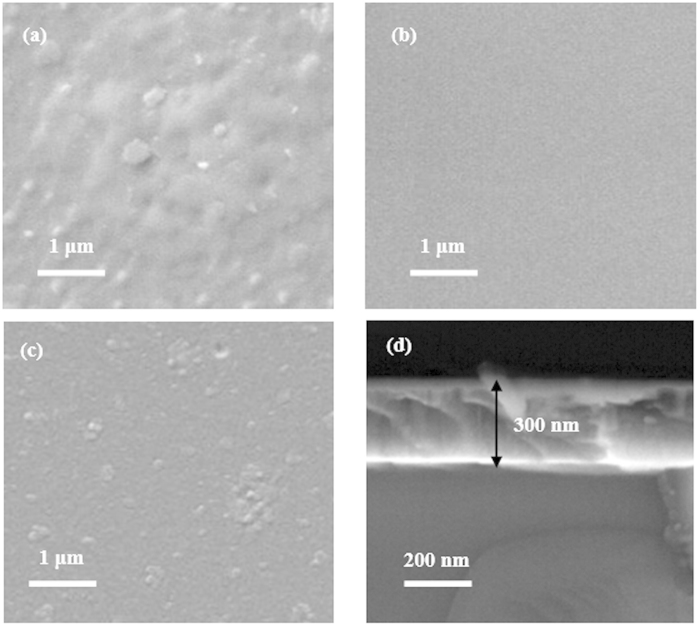
SEM images for ~300 nm-thick AlN films grown on Si (111) substrates at (**a**) 850, (**b**) 750, and (**c**) 700 °C. (**d**) The cross-sectional SEM of AlN films grown on Si (111) substrates at 750 °C for 90 min.

**Figure 4:**
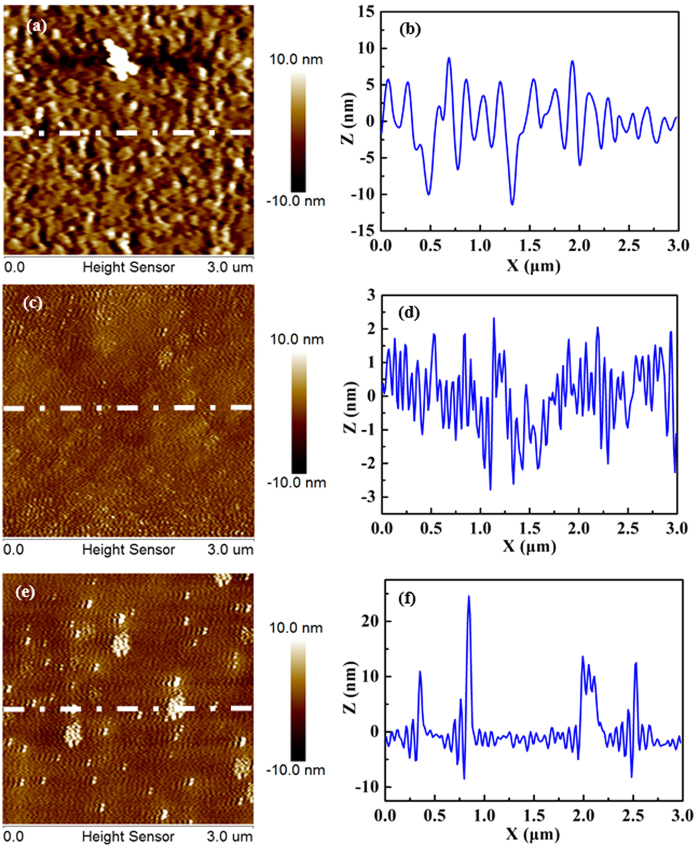
(**a**) AFM images for ~300 nm-thick AlN films grown on Si (111) substrates at 850 °C and (**b**) the height profiles along a straight dashed line on the surface. (**c**) AFM images for ~300 nm-thick AlN films grown on Si (111) substrates at 750 °C and (**d**) the height profiles along a straight dashed line on the surface. (**e**) AFM images for ~300 nm-thick AlN films grown on Si (111) substrates at 700 °C and (**f**) the height profiles along a straight dashed line on the surface.

**Figure 5:**
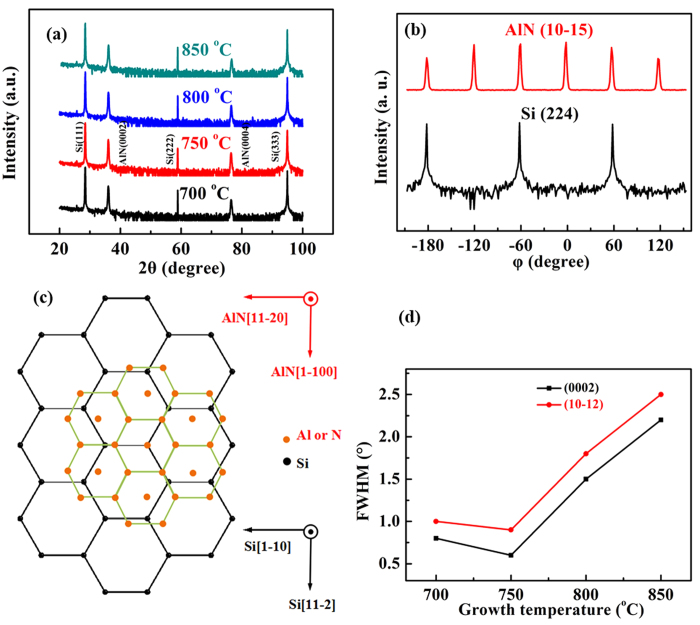
(**a**) Temperature dependence of 2*θ*-ω scan for ~300 nm-thick AlN films grown on Si(111) substrates at temperatures ranging from 700 to 850 °C. (**b**) Typical *φ* scans of Si(224) and AlN(10–15). (**c**) Schematic structure in-plane relationship between AlN and Si. (**d**) Temperature dependence of FWHMs for ~300 nm-thick AlN films grown on Si(111) substrates at temperatures ranging from 700 to 850 °C.

**Figure 6:**
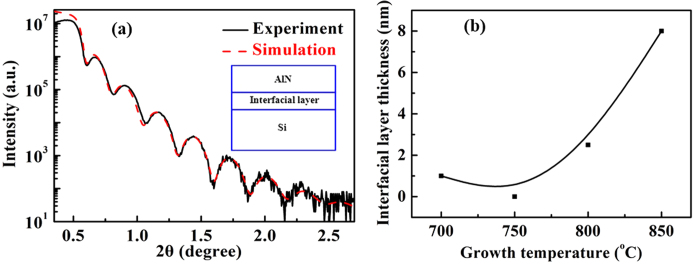
(**a**) GIXR and its simulated curves for AlN films grown on Si(111) substrates. (**b**) Temperature dependence of interfacial layer thickness for AlN AlN films grown on Si(111) substrates.

**Figure 7:**
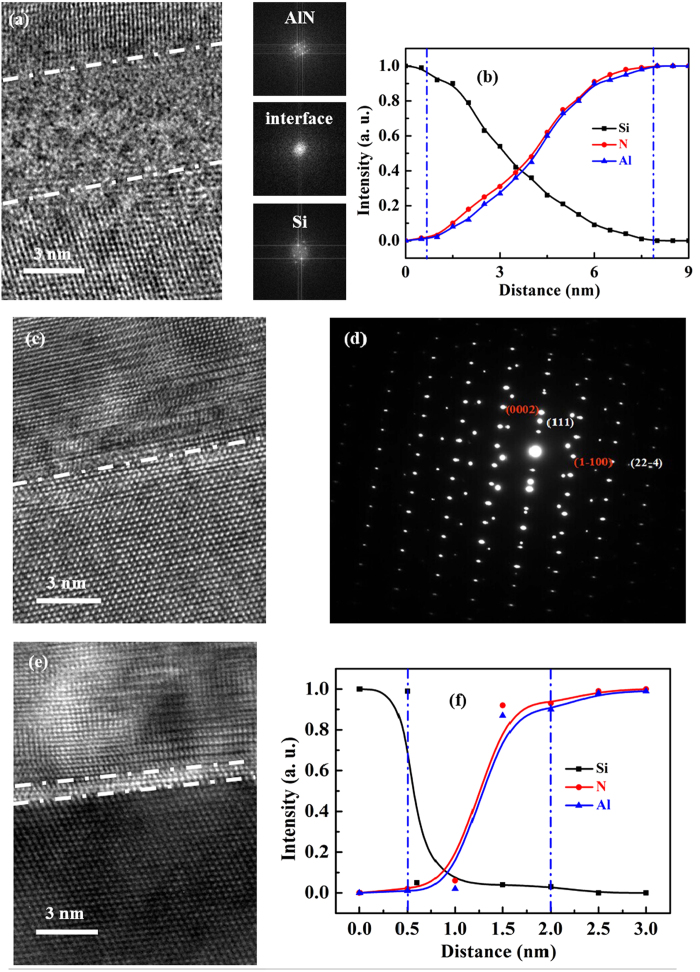
(**a**) Cross-sectional TEM image for AlN films grown on Si substrates at 850 °C, and (**b**) the EELS measurement across the corresponding AlN/Si hetero-interfaces. (**c**) Cross-sectional TEM image for AlN films grown on Si substrates at 750 °C, and (**d**) the SAED patterns across the corresponding AlN/Si hetero-interfaces. (**e**) Cross-sectional TEM image for AlN films grown on Si substrates at 700 °C, and (**f**) the EELS measurement across the corresponding AlN/Si hetero-interfaces.

**Figure 8:**
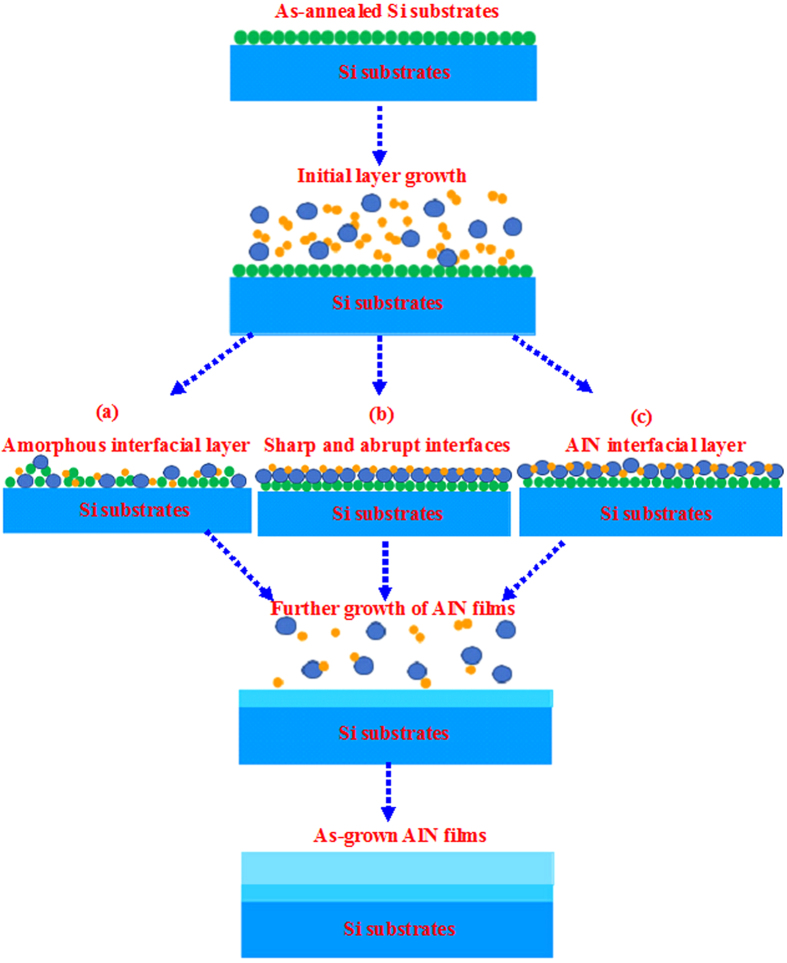
Schematic diagrams for the AlN films grown on Si substrates at (**a**) 850, (**b**) 750, and (**c**) 700 °C, respectively.

## References

[b1] Liu L. & Edgar J. H. Substrates for gallium nitride epitaxy. Mater. Sci. Eng. R 37, 61–127 (2002).

[b2] Huang K. Top- and bottom-emission-enhanced electroluminescence of deep-UV light-emitting diodes induced by localised surface plasmons. Sci. Rep. 4, 4380 (2014). 24625660 10.1038/srep04380 PMC3953721

[b3] Yang W. . High density GaN/AlN quantum dots for deep UV LED with high quantum efficiency and temperature stability. Sci. Rep. 4, 5166 (2014). 24898569 10.1038/srep05166 PMC4046137

[b4] Reitmeier Z. J. . Surface and defect microstructure of GaN and AlN layers grown on hydrogen-etched 6H–SiC(0001) substrates. Acta Mater. 58, 2165–2175 (2010).

[b5] Lin K. L. . MOVPE high quality GaN film grown on Si (111) substrates using a multilayer AlN buffer. Phys. Status Solidi C 5, 1536–1538 (2008).

[b6] Zhu D., Wallis D. J. & Humphreys C. J. Prospects of III-nitride optoelectronics grown on Si. Rep. Prog. Phys. 76, 106501 (2013). 24088511 10.1088/0034-4885/76/10/106501

[b7] Nikishin S. . High quality AlN for deep UV photodetectors. Appl. Phys. Lett. 95, 054101 (2009).

[b8] Sung C. C., Chiang Y. F., Ro R., Lee R. & Wu S. Effects of conducting layers on surface acoustic wave in AlN films on diamond. J. Appl. Phys. 106, 124905 (2009).

[b9] Takagaki Y. . Superhigh-frequency surface-acoustic-wave transducers using AlN layers grown on SiC substrates. Appl. Phys. Lett. 81, 2538–2540 (2002).

[b10] Zhou C. J. . Visible-light photoresponse of AlN-based film bulk acoustic wave resonator. Appl. Phys. Lett. 102, 191914 (2013).

[b11] Yang H., Wang W., Liu Z., Yang W. & Li G. Epitaxial growth mechanism of pulsed laser deposited AlN films on Si (111) substrates. CrystEngComm 16, 3148–3154 (2014).

[b12] Yang H., Wang W., Liu Z. & Li G. Homogeneous epitaxial growth of AlN single-crystalline films on 2 inch-diameter Si (111) substrates by pulsed laser deposition. CrystEngComm 15, 7171–7176 (2013).

[b13] Kioseoglou J., Lotsari A., Kalesaki E. & Dimitrakopulos G. P. Interfaces between nonpolar and semipolar III-nitride semiconductor orientations: Structure and defects. J. Appl. Phys. 111, 033507 (2012).

[b14] Radtke G., Couillard M., Botton G. A., Zhu D. & Humphreys C. J. Structure and chemistry of the Si(111)/AlN interface. Appl. Phys. Lett. 100, 011910 (2012).

[b15] Bourret A., Barski A., Rouvière J. L., Renaud G. & Barbier A. Growth of aluminum nitride on (111) silicon: Microstructure and interface structure. J. Appl. Phys. 83, 2003–2009 (1998).

[b16] Wang W. . Epitaxial growth of high quality AlN films on metallic aluminum substrates. CrystEngComm 16, 4100–4107 (2014).

[b17] Wang W. . Effect of nitrogen pressure on the properties of AlN films grown on nitrided Al(111) substrates by pulsed laser deposition. Mater. Lett. 129, 39–42 (2014).

[b18] Feiler D., Williams R. S., Talin A. A., Yoon H. & Goorsky M. S. Pulsed laser deposition of epitaxial AlN, GaN, and InN thin films on sapphire(0001). J. Cryst, Growth 171, 12–20 (1997).

[b19] Li G., Kim T.-W., Inoue S., Okamoto K. & Fujioka H. Epitaxial growth of single-crystalline AlN films on tungsten substrate. Appl. Phys. Lett. 89, 241905 (2006).

[b20] Kim T.-W., Matsuki N., Ohta J. & Fujioka H. Epitaxial growth of AlN on single-crystal Ni(111) substrates. Appl. Phys. Lett. 88, 121916 (2006).

[b21] Wang W. . Epitaxial growth of high-quality AlN films on metallic nickel substrates by pulsed laser deposition. RSC Adv. 4, 27399–27403 (2014).

[b22] Gaire C., Tang F. & Wang G. C. *In-situ* reflection high-energy electron diffraction study of epitaxial growth of Cu on NaCl (100) under oblique angle vapor deposition. Thin Solid Films 517, 4509–4514 (2009).

[b23] Sawadaishi M., Taguchi S., Sasaya K. & Hond T. Nitridation of (111)Al substrates for GaN growth by molecular beam epitaxy. J. Cryst. Growth 311, 1994–1996 (2009).

[b24] Yang H., Wang W., Liu Z. & Li G. Epitaxial growth of 2 inch diameter homogeneous AlN single-crystalline films by pulsed laser deposition. J. Phys. D: Appl. Phys. 46, 105101 (2006).

[b25] Martin Y. Williams C. C. & Wickramasinghe H. K. Atomic force microscope–force mapping and profiling on a sub 100Å scale. J. Appl. Phys. 61, 4723–4729 (1987).

[b26] Feng Y. X. . Significant quality improvement of GaN on Si(111) upon formation of an AlN defective layer. CrystEngComm 16, 7525–7528 (2014).

[b27] Moram M. A. & Vickers M. E. X-ray diffraction of III-nitrides. Rep. Prog. Phys. 72, 036502 (2009).

[b28] Chen X. W., Jia C. H., Chen Y. H., Wang H. T. & Zhang W. F. Epitaxial growth and optical properties of Al- and N-polar AlN films by laser molecular beam epitaxy. J. Phys. D: Appl. Phys. 47, 125303 (2014).

[b29] Birkholz M. Thin Film Analysis by X-Ray Scattering (WILEY-VCH Verlag GmbH & Co. KGaA, 2006).

[b30] Dadgar A. . Epitaxy of GaN on silicon-impact of symmetry and surface reconstruction. New J. Phys. 9, 389 (2007).

[b31] Wang W. . Epitaxial growth of homogeneous single-crystalline AlN films on single-crystalline Cu (111) substrates. Appl. Surf. Sci. 294, 1–8 (2014).

[b32] Wang W. . Achieve high-quality InGaN/GaN multiple quantum wells on La_0.3_Sr_1.7_AlTaO_6_ substrates. Mater. Lett. 128, 27–30 (2014).

[b33] Wang W., Yang H. & Li G. Achieve high-quality GaN films on La_0.3_Sr_1.7_AlTaO_6_ (LSAT) substrates by low-temperature molecular beam Epitaxy. CrystEngComm 15, 2669–2674 (2013).

[b34] Egerton R. F. Electron Energy-Loss Spectroscopy in the Electron Microscope (Springer, 2011)

[b35] Ding Y. & Wang Z. L. Electron energy-loss spectroscopy study of ZnO nanobelts. J. Electron Microsc. 54, 287–291 (2005). 10.1093/jmicro/dfi039 16123068

[b36] Gao F. & Li G. Quality-enhanced In_0.3_Ga_0.7_As film grown on GaAs substrate with an ultrathin amorphous In_0.6_Ga_0.4_As buffer layer. Appl. Phys. Lett. 104, 042104 (2014).

[b37] Moram M. A. . On the origin of threading dislocations in GaN films. J. Appl. Phys. 106, 073513 (2009).

[b38] Oliver R. A. . Microstructural origins of localization in InGaN quantum wells. J. Phys. D: Appl. Phys. 43, 354003 (2010).

[b39] Coulombwall A. Growth and applications of Group III-nitrides. J. Phys. D: Appl. Phys. 31, 2653–2710 (1998).

[b40] Widmann F., Feuillet G., Daudin B. & Rouvière J. L. Low temperature sapphire nitridation: A clue to optimize GaN layers grown by molecular beam epitaxy. J. Appl. Phys. 85, 1550–1555 (1999).

[b41] Wang W. . Epitaxial growth and characterization of high-quality aluminum films on sapphire substrates by molecular beam epitaxy. CrystEngComm 16, 7626–7632 (2014).

[b42] Liu R., Ponce F. A., Dadgar A. & Krost A. Atomic arrangement at the AlN/Si (111) interface. Appl. Phys. Lett. 83, 860–862 (2003).

